# Competitive Regulation of E-Cadherin JuxtaMembrane Domain Degradation by p120-Catenin Binding and Hakai-Mediated Ubiquitination

**DOI:** 10.1371/journal.pone.0037476

**Published:** 2012-05-31

**Authors:** Andrea Hartsock, W. James Nelson

**Affiliations:** 1 Department of Molecular and Cellular Physiology, Stanford University, Stanford, California, United States of America; 2 Department of Biology, Stanford University, Stanford, California, United States of America; University of Washington, United States of America

## Abstract

p120-Catenin binding to, and Hakai-mediated ubiquitination of the E-cadherin juxtamembrane domain (JMD) are thought to be involved in regulating E-cadherin internalization and degradation. However, the relationship between these two pathways is not understood. We targeted the E-cadherin JMD to mitochondria (WT-JMD) to isolate this domain from the plasma membrane and internalization, and to examine protein modifications and degradation. WT-JMD localized to mitochondria, but did not accumulate there except when proteasome activity was inhibited. We found WT-JMD was ubiquitinated, and arginine substitution of lysines at position 5 (K5R) and 83 (K83R) resulted in the stable accumulation of mutant JMD at mitochondria. p120-Catenin did not localize, or bind to WT-JMD even upon proteasome inhibition, whereas the K5,83R-JMD mutant bound and localized p120-catenin to mitochondria. Mutation of the p120-catenin binding site in combination with these lysine mutations inhibited p120-catenin binding, but did not decrease JMD stability or its accumulation at mitochondria. Thus, increased stability of JMD lysine mutants was due to inhibition of ubiquitination and not to p120-catenin binding. Finally, mutation of these critical lysines in full length E-cadherin had similar effects on protein stability as WT-JMD. Our results indicate that ubiquitination of the JMD inhibits p120-catenin binding, and targets E-cadherin for degradation.

## Introduction

The level of membrane proteins at the plasma membrane is regulated by post-translational modifications including phosphorylation and ubiquitination. For example, Epithelial Growth Factor Receptor (EGFR) is ubiquitinated and internalized leading to the recycling of EGFR and/or the degradation of both receptor and its ligand (for review see [1]). In addition, both tyrosine [2], [3], [4] and non-tyrosine kinase receptors [5] are ubiquitinated and degraded in a proteasome-dependent manner. Ubiquitination and proteasome degradation of transmembrane receptors may depend on ligand binding in the case of Growth Hormone Receptor [4] and Interleukin-5 [3], or not in the case of Met Tyrosine Kinase Receptor [2] and Interleukin-9 and -2 [5]. Plasma membrane levels of the epithelial Na^+^-channel (ENaC) are regulated by ubiquitination and internalization [6], but it is unclear whether levels of non-receptor transmembrane proteins are also controlled by subsequent degradation by the proteasome.

Tissue development is a dynamic process requiring stages of stabilization and loss of cell-cell adhesion [7]. E-cadherin, a member of the Ca^2+^-dependent cadherin superfamily of cell-cell adhesion proteins, has multiple functions at the plasma membrane including initiation and stabilization of cell-cell adhesion, regulation of the actin cytoskeleton, intracellular signaling and cell polarization [7]. E-cadherin function and organization require interactions with catenin family members, β-catenin and α-catenin, which are involved in linkages to the actin cytoskeleton, and p120-catenin which regulates E-cadherin localization to the plasma membrane [8].

p120-Catenin was first identified as a substrate for Src tyrosine kinase [9] and later defined as a member of the catenin family based on sequence homology with the armadillo domain of β-catenin [10]. The p120-catenin binding site on E-cadherin is localized in a short amino acid sequence of 93 amino acids (the JuxtaMembrane Domain (JMD)) that contains the octapeptide sequence YDEEGGGE [11]. The JMD is required for E-cadherin stability and cell-cell adhesion [12]. Binding of p120-catenin to the JMD is proposed to prevent E-cadherin from being internalized and degraded [13], [14], [15], or recycle internalized cadherin back to the plasma membrane [16]. Hakai, an E3-ubiquitin ligase, also binds the JMD in a Src phosphorylation-dependent manner, and increases E-cadherin ubiquitination and internalization [17]. It is not known whether ubiquitination of the JMD and p120-catenin binding to the JMD are independent or coupled events. In addition, it is not known if ubiquitination of the JMD or p120-catenin binding to the JMD is involved in E-cadherin degradation since it has been difficult to uncouple roles of ubiquitination and p120-catenin binding in E-cadherin/JMD internalization and/or degradation.

To isolate mechanisms involved in JMD-mediated E-cadherin degradation, we targeted E-cadherin JMD to mitochondria to directly examine protein-protein interactions and JMD modifications. This method allowed us to establish an *in vivo* protein degradation assay to analyze the stability of JMD, JMD ubiquitination, and JMD/p120-catenin interactions independent of cell-cell adhesion and cell migration. We verified results from this *in vivo* protein degradation assay with studies of full-length E-cadherin stabilization at the plasma membrane. Our results indicate that the JMD regulates the degradation of E-cadherin by the competition between binding of p120-catenin and ubiquitination.

## Results

### E-Cadherin JMD Level is Regulated by Proteasomal Degradation

The plasma membrane at cell-cell contacts is crowded with proteins that make it difficult to discriminate binding of specific protein complexes, and/or identify post-translational modifications. Previous studies have utilized mis-targeting of proteins to other intracellular sites to test protein functions [18], [19]. We used this method to spatially isolate the E-cadherin JMD from the plasma membrane in order to study protein binding and modifications involved in E-cadherin JMD regulation.

**Figure 1:**
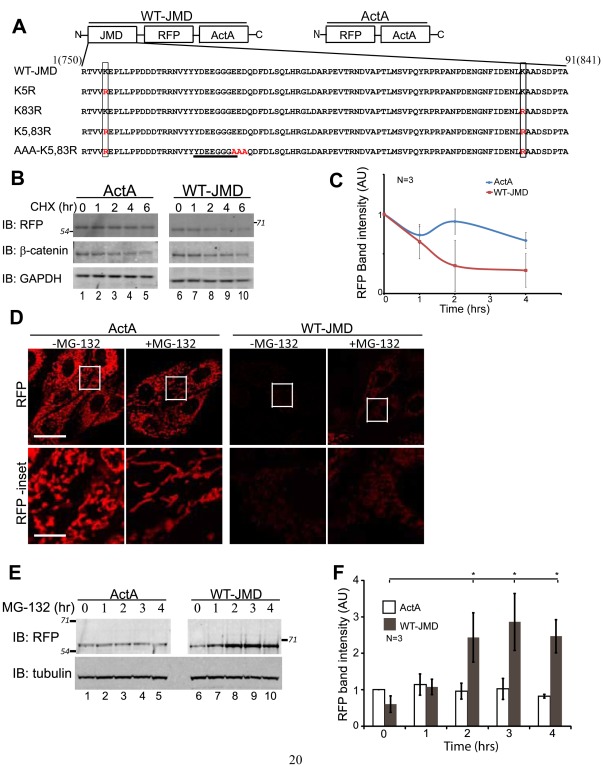
E-cadherin JMD Level is Regulated by Proteasomal Degradation. (A) Top: schematic representation of the Juxtamembrane domain (JMD) expression construct (left) and ActA control construct (right). Bottom: mouse E-cadherin JMD sequences aligned to demonstrate differences in mutations utilized in this study. Lysine to arginine mutations are denoted by rectangular boxes (K#R and Red “R”). Red AAA denotes mutations to abolish E-cadherin JMD/p120-catenin binding. E-cadherin octapeptide sequence required for binding p120-catenin is underlined. Numbers above the sequence represent the amino acid position number, and the corresponding amino acid position in murine E-cadherin sequence in parenthesis. (B) MDCK cells stably expressing ActA or WT-JMD were incubated for 6 hours with cycloheximide to determine protein turnover. β-catenin used as a degradation control and GAPDH as a loading control. Number(s) on the side of gels represent apparent molecular weights of protein standards (× 10^∧^3). (C) Quantification of immunoblots of MDCK cells stably expressing ActA or WT-JMD in cycloheximide time course experiment (see B). Band intensities normalized to GAPDH to account for the reduction in overall protein levels. Results were averaged (+/− standard error of the mean (s.e.m.)) from 3 different experiments (N = 3). (D) Fluorescence images of MDCK cells transiently expressing RFP (ActA) or E-cadherin JMD RFP fusion protein (WT-JMD) targeted to the mitochondria; boxed regions in “RFP” are shown below at a higher magnification in “RFP-inset.” Scale bar is 25 µm in 100X images, and 5 µm in insets. (E) WT-JMD levels increase 4-fold upon proteasome inhibition. WT-JMD migrated with an apparent molecular weight that was ∼15kDa greater than ActA. Number(s) on the side of the gels are the apparent molecular weights of protein standards (× 10^∧^3). (F) Quantification of immunoblot RFP protein levels averaged (+/− s.e.m.) from 3 different experiments; *p≤0.034.

The E-cadherin JMD, comprising 91 amino acids that include the octapeptide domain required for p120-catenin binding (Figure 1A), was targeted to mitochondria in MDCK cells using the ActA peptide [19] coupled to monomeric RFP as a fluorescent read-out of expression and localization (WT-JMD; Figure 1A- top left). A chimera of monomeric RFP and the ActA peptide (ActA) was used as a control (ActA; Figure 1A- top right).

Expression of WT-JMD or ActA did not affect the localization of E-cadherin to cell-cell junctions, ZO-1 to the tight junction, vinculin to focal adhesions, nor the microtubule or actin networks (Figure S1A). Expression of either construct also did not affect epithelial sheet organization and migration compared to parental MDCK cells (Figure S1B, C). Therefore, targeting of the E-cadherin JMD to mitochondria did not appear to affect normal cell-cell adhesion, epithelial sheet migration, or the organization of the cytoskeleton.

To examine protein turnover, WT-JMD and ActA stable cell lines were created using MDCK cells (see Materials and Methods *Stable Cell Lines*; [20]). Both WT-JMD and ActA stable cell lines were incubated with cycloheximide (CHX) for up to 6 hours to inhibit further protein synthesis and allow analysis of the rate of degradation of existing proteins. Levels of β-catenin were used as a control for comparison with known levels of protein degradation [21]. At 6 hours, the level of ActA was ∼60% of the starting level, while the level of WT-JMD was ∼30%. β-Catenin levels decreased ∼50% (Figure 1B, C) as shown before.

WT-JMD and ActA RFP localized to mitochondria based on co-localization with MitoTracker (Figure S2) and mitochondrial heat shock protein 70 (A. Hartsock, unpublished results). However, in transiently expressing cells and three independently cloned stable cell lines the level of ActA RFP fluorescence localized at mitochondria was much greater than WT-JMD based on RFP fluorescence (Figure S2) and immunofluorescence with an anti-RFP antibody (Figure 1D).

To determine if the low level of WT-JMD fluorescence compared to ActA was due to degradation of WT-JMD, we inhibited the proteasome with MG-132 for up to 4 hours. We detected increased WT-JMD RFP fluorescence (Figure 1D). For western blot analysis, we used a lower expressing ActA stable cell line to analyze RFP levels comparable to WT-JMD, which allowed us to observe changes in ActA levels which may not have been obvious in the higher expressing cell lines. Western blotting of RFP levels revealed a 4-fold increase in WT-JMD protein in the presence of MG-132 (*p  = 0.018; Figure 1E, F). There was not a statistically significant increase in RFP upon proteasome inhibition in ActA stable cells (Figure 1E, F). Note that ActA protein levels did not increase upon proteasome inhibition in higher expressing stable cell lines (Figure 5B). Thus, the low level of WT-JMD compared to ActA was due to WT-JMD-degradation by the proteasome.

### E-Cadherin Juxtamembrane Domain is Ubiquitinated

**Figure 2:**
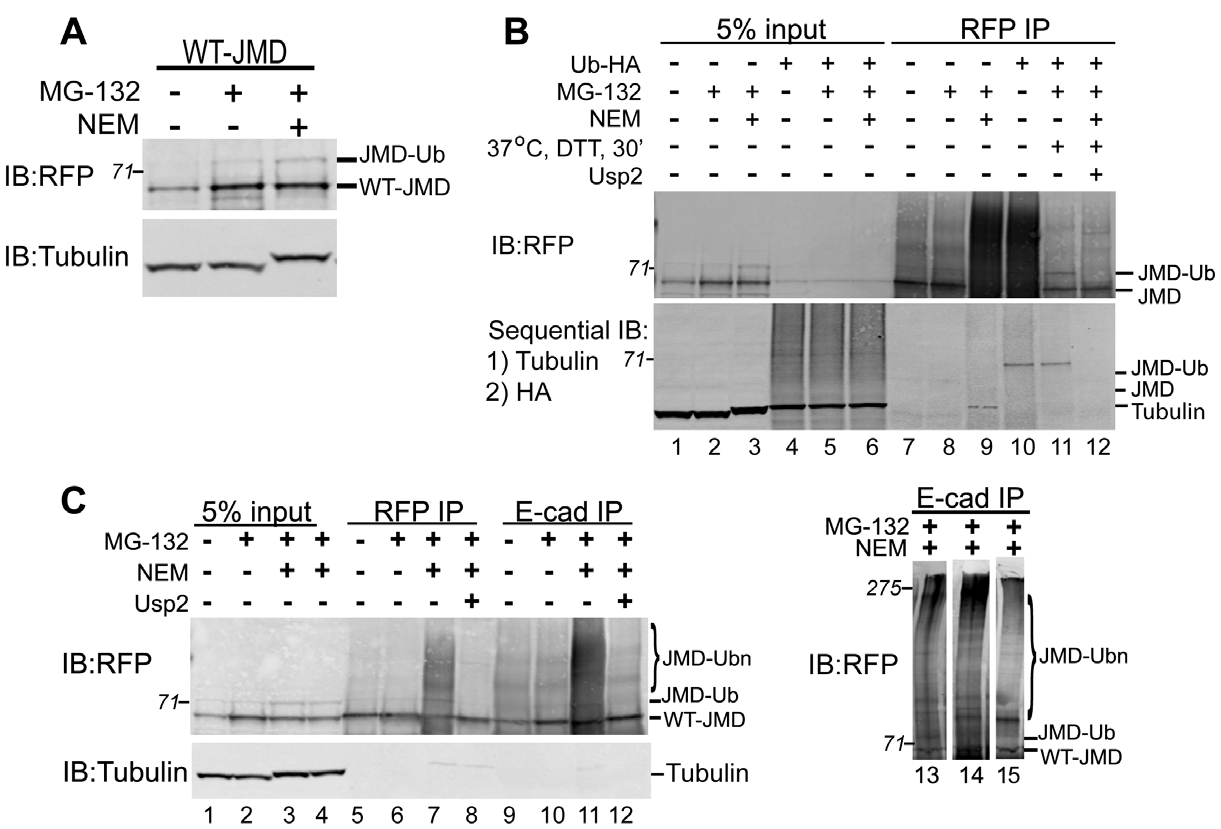
E-cadherin JMD is Ubiquitinated. (A) Lysates of WT-JMD stable cell lines under normal conditions, upon proteasome inhibition (MG-132), or inhibition of deubiquitinating enzymes (NEM). Immunoblot (IB) for RFP shows a slower migrating band (JMD-Ub) in the presence of MG-132 and NEM. (B) In a separate experiment MDCK cells stably expressing WT-JMD were transiently transfected with Ub-HA where indicated. Cells were extracted and RFP immunoprecipitations were preformed under normal conditions, upon proteasome inhibition, NEM treatment (to inhibit de-ubiquitinating enzymes), addition of the deubiquitinating enzyme Usp2, or mock transfections. DTT was added to the immunoprecipitates after the final was of Protein A beads but prior to the addition of Usp2, where indicated, to neutralize residual NEM. Immunoblots (IB) were performed for RFP using a rabbit polyclonal antibody, followed by a sequential immunoblotting with antibodies specific for: 1) tubulin; and 2) HA. The slowest migrating band (marked as JMD-Ub) that appears in the presence of NEM is positive for RFP and HA (lanes 10 and 11). HC denotes IgG heavy chain. Number(s) on the side of the gels are the apparent molecular weights of protein standards (× 10^∧^3). (C) Extracts from MDCK cells stably expressing WT-JMD were immunoprecipitated (IP) for RFP and E-cadherin under normal conditions, upon proteasome inhibition, NEM treatment (to inhibit de-ubiquitinating enzymes), or addition of the de-ubiquitinating enzyme Usp2. DTT was added to the immunoprecipitates after the final wash of Protein A beads but prior to the addition of Usp2, where indicated, to neutralize residual NEM. A slow migrating band (lane 15) and protein smear (lanes 11, 13, and 14) appear in E-cadherin immunoprecipitates in the presence of NEM, and collapse upon incubation with Usp2 (similar to RFP immunoprecipitates). RFP immunoblots in lanes 11, 13, 14 & 15 show that the slower migrating band does migrates at different molecular weights indicating variable levels of JMD ubiquitination. Number(s) on the side of the blots are the apparent molecular weights of protein standards (× 10^∧^3). Data are representative of 3 different experiments.

Since the level of WT-JMD increased when the proteasome was inhibited (Figure 1D-F), we tested whether WT-JMD was ubiquitinated (JMD-Ub). Western blot analysis of WT-JMD cell extracts did not reveal a slower migrating band or high molecular weight smear corresponding to the addition of ubiquitin(s). It is possible that WT-JMD was ubiquitinated, but deubiquitinating enzymes in the cell extract removed ubiquitin. Therefore, proteins were extracted from cells in the presence of N-ethylameliamide (NEM), a non-specific inhibitor of de-ubiquitination [22]. In the presence, but not in the absence of NEM, we detected WT-JMD and a slower migrating band positive for RFP by Western blotting (Figure 2A); this slower migrating protein band had an apparent increase in molecular weight of ∼7 kDa compared to WT-JMD, consistent with the addition of one ubiquitin. We did not detect an equivalent slower migrating band in ActA from control cell lysates or ActA immunoprecipitates (Figure 5B; lane 3).

To test directly whether WT-JMD was ubiquitinated, ubiquitin-HA (Ub-HA) was transiently expressed in WT-JMD stable cells. Total proteins were extracted under normal conditions in both mock and Ub-HA transfections, upon proteasome inhibition, or NEM treatment (to inhibit de-ubiquitinating enzymes). Sequential immunoblotting were performed using mouse monoclonal antibodies for tubulin, and then HA. A protein smear was present throughout the lane in whole cell lysates of WT-JMD cells transfected with Ub-HA only after immunoblotting for HA (Figure 2B, lanes 4–6). WT-JMD immuno-precipitates with RFP antibody transiently expressing Ub-HA revealed a slower migrating band that reacted with both rabbit polyclonal RFP and mouse monoclonal HA antibodies by western blotting (JMD-Ub; Figure 2B, lane 10 and 11). This protein band was only detected on the sequential immunoblot with the mouse monoclonal HA antibody. Importantly, when RFP was immunoprecipitated in the presence of NEM and then treated with the deubiquitinating enzyme Usp2 the slower migrating band was lost in both RFP and HA western blots (Figure 2C, lane 12), confirming that “JMD-Ub” was ubiquitinated. Note that DTT was added to the immunoprecipitates after the final wash but prior to the addition of Usp2 to neutralize residual NEM. RFP immunoprecipitates subjected to DTT and incubation without the addition of Usp2 did not result in loss of the slower migrating band (JMD-Ub; Figure 2B lanes 10 and 11) further confirming JMD-Ub was ubiquitinated.

**Figure 3:**
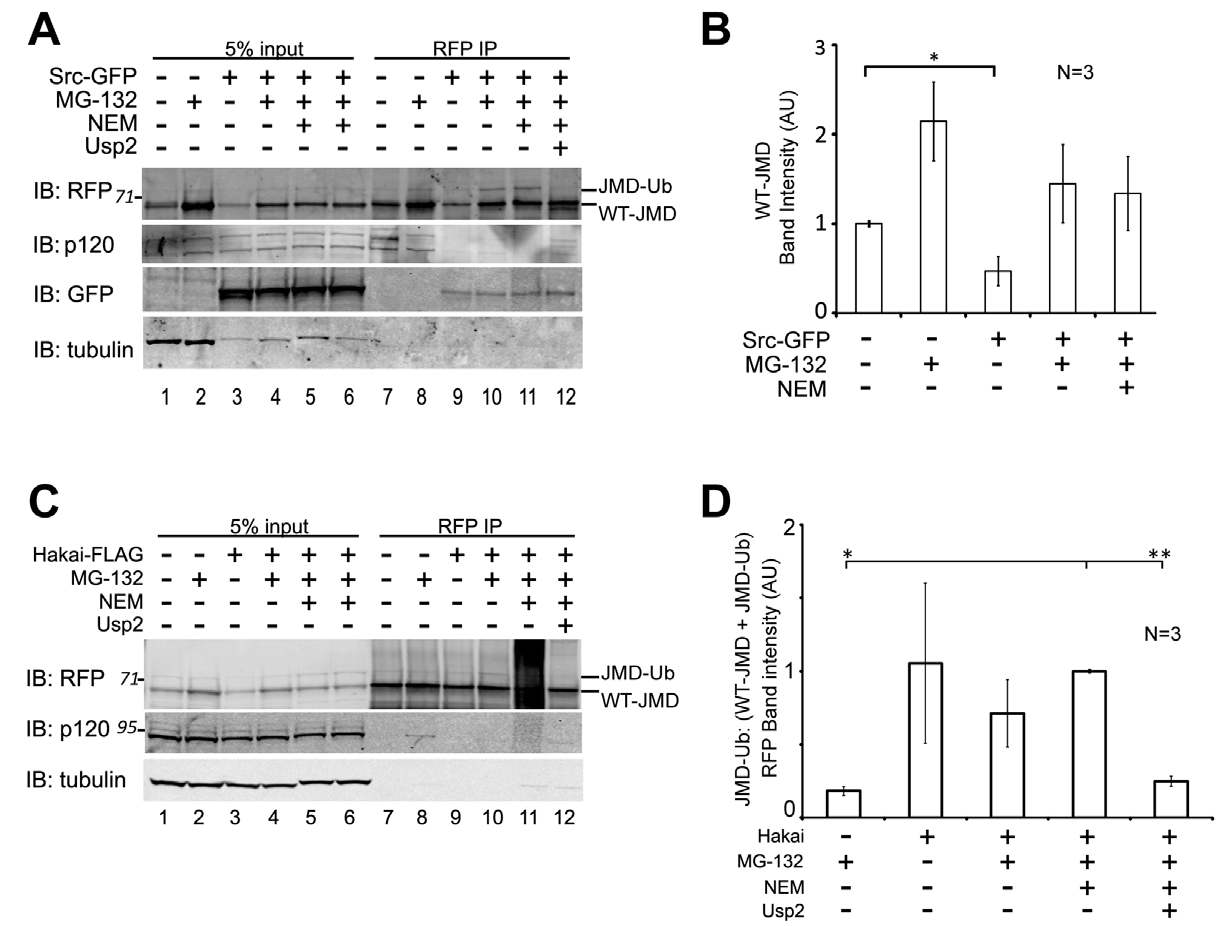
Expression of Src-GFP or Hakai-FLAG increase JMD degradation and ubiquitination. (A) MDCK cells stably expressing WT-JMD were transiently transfected with Src-GFP. Cells were extracted and RFP immunoprecipitations (IP) were performed under normal conditions, upon proteasome inhibition, NEM treatment (to inhibit de-ubiquitinating enzymes), or addition of the de-ubiquitinating enzyme Usp2. De-ubiquitinating enzyme Usp2 (with DTT) was added, where indicated, to immunoprecipitates after the last step of washing Protein A beads. Membranes were immunoblotted (IB) for RFP, GFP, p120-catenin, and tubulin. Number(s) on the side of the gels are the apparent molecular weights of protein standards (× 10^∧^3). (B) Quantification of immunoblots for WT-JMD upon expression of Src-GFP in WT-JMD stable cells. RFP WT-JMD band intensities from immunoprecipitates were normalized to tubulin to account for reduction in overall protein levels as a result of transfections. *p<0.05. Results are averaged from 3 different experiments (+/− s.e.m.). (C) MDCK cells stably expressing WT-JMD were transiently transfected with Hakai-FLAG. Cells were extracted and RFP immunoprecipitations (IP) were preformed under normal conditions, upon proteasome inhibition, NEM treatment (to inhibit de-ubiquitinating enzymes), or addition of the de-ubiquitinating enzyme Usp2. De-ubiquitinating enzyme Usp2 (with DTT) was added, where indicated, to immunoprecipitates after the last step of washing Protein A beads. Immunoblots (IB) were performed for RFP, p120-catenin and tubulin. Numbers on the side of the gels are the apparent molecular weights of protein standards (× 10^∧^3). (D) Quantification of the ratio of JMD-Ub level to total mitochondrial targeted JMD protein (WT-JMD + JMD-Ub) upon expression of Hakai-FLAG in WT-JMD stable cells. Band intensities from immunoprecipitates were normalized to tubulin. Results are averaged from 3 independent experiments (+/− s.e.m.). *p<0.01, **p<0.001.

We also used a rabbit polyclonal antibody raised to the cytoplasmic domain of E-cadherin to immunoprecipitate WT-JMD. Western blots of E-cadherin immunoprecipitates confirmed an increase in the level of WT-JMD upon proteasome inhibition (Figure 2C, lanes 9, 10). Furthermore, a RFP-positive, protein smear was present only when extractions were performed in the presence of NEM (Figure 2C, lane 11). The slower migrating WT-JMD band migrated at different molecular weights (JMD-Ub_n_) in four different experiments as either a single band (Figure 2C, lane 15), or more commonly a protein smear (Figure 2C, lanes 11, 13, 14). The slower migrating protein band and the smear were lost upon incubation of immunoprecipitates with the deubiquitinating enzyme Usp2 (Figure 2C, lanes 8 and 12; A. Hartsock, unpublished results). While we observed that tubulin co-immunoprecipitated with JMD when cells were treated with both MG-132 and NEM, we feel this is an artifact of NEM treatment as this only appears upon the addition of NEM (Figure 3A and 3C) and in control cell immunoprecipitations under the same conditions (Figure 5B). Thus, E-cadherin immunoprecipitations indicate that WT-JMD can be poly-ubiquitinated (Figure 2C).

### Over-expression of Src or Hakai Increases JMD Degradation

Src kinase phosphorylates E-cadherin cytoplasmic domain, stabilizing binding of the E3 ligase Hakai to E-cadherin which ubiquitinates the E-cadherin cytoplasmic domain [17]. However, it is unknown whether p120-catenin binding to E-cadherin JMD is affected under these conditions. Therefore, we over-expressed either Src kinase (Src-GFP) or Hakai (Hakai-FLAG) in the presence or absence of proteasomal inhibition and/or inhibition of deubiquitination (NEM treatment), and examined WT-JMD levels and p120-catenin binding.

Over-expression of Src-GFP decreased the amount of WT-JMD by 50% compared to mock transfections (Figure 3A, lanes 1, 7; Figure 3B; *p  = 0.042). Upon proteasome inhibition, the amount of WT-JMD increased 2-fold in mock transfected cells and 3-fold in Src-GFP expressing cells (Figure 3A, compare lanes 1 vs. 2, 3 vs. 4; Figure 3B). A slower migrating band similar to the ubiquitinated protein band (Figure 2B) was observed in Src-GFP expressing cells upon proteasome inhibition and NEM treatment (Figure 3A, lanes 10–11). This slower migrating protein was not present in immunoprecipitates incubated with the de-ubiquitinating enzyme Usp2 (Figure 3A, lane 12). Analysis of the level of JMD-Ub compared to total mitochondrial targeted JMD protein (WT-JMD + JMD-Ub) demonstrated that upon Src-GFP expression there were equivalent levels of JMD-Ub that were not statistically different. However, the level of JMD-Ub was statistically greater compared to mock transfected cells treated with a proteasome inhibitor (p = 0.05). Therefore, we conclude that JMD-Ub levels in Src-GFP expressing cells were increased relative to total mitochondrial targeted JMD protein.

The level of ubiquitinated WT-JMD (JMD-Ub) did not appear to change when Src-GFP was over-expressed (Figure 3A, lanes 7–10; Figure 3B), and was unaffected by proteasome inhibition compared to the mock transfected control (Figure 3A, lanes 8, 10; Figure 3B). However, western blots of p120-catenin showed a clear decrease in level of the two p120-catenin isoforms that co-immunoprecipiated with WT-JMD when Src-GFP was over-expressed (Figure 3A, lanes 9–11).

Expression of Hakai-FLAG in cells stably expressing WT-JMD affected the level of WT-JMD ubiquitination. Hakai-FLAG expression in WT-JMD cells decreased the level of WT-JMD compared to mock transfections (Figure 3C, lanes 1, 3). The slower migrating WT-JMD band in cell lysates expressing Hakai-FLAG had the same apparent molecular weight as the slower migrating WT-JMD (JMD-Ub) that accumulated upon inhibition of deubiquitinating enzymes in mock transfected cells (Figure 3C, lanes 9–11), but was absent from immunoprecipitates incubated with the deubiquitinating enzyme Usp2 (Figure 3C, lane 12). Similar analysis of the level of JMD-Ub as a ratio to total mitochondrial targeted JMD protein (WT-JMD + JMD-Ub) demonstrated that upon Hakai-FLAG expression there were equivalent levels of JMD-Ub and that these levels were significantly increased in comparison to mock transfected cells upon proteasome inhibition (Figure 3D; *p<0.01, **p<0.001). This analysis allowed us to determine that JMD-Ub levels in Hakai-FLAG expressing cells were increased relative to total mitochondrial targeted JMD protein.

Western blots of p120-catenin showed a reduction in the amount of p120-catenin bound to WT-JMD upon proteasome inhibition in Hakai-FLAG expressing cells compared to the small amount present in mock transfections under similar conditions (Figure 3C, lanes 8, 10). p120-Catenin was not bound to WT-JMD when Hakai-FLAG was over-expressed and cell lysates were treated with NEM, or immunoprecipitates were treated with the deubiquitinating enzyme Usp2 (Figure 3C, lanes 11–12). It should be noted that due to antibody cross-reactivity two different p120-catenin antibodies were used in this study. While the rabbit p120-catenin antibody recognized two isoforms of p120-catenin (see Figure 3A, lanes 1–6) the mouse p120-catenin antibody recognizes three proteins (Figure 3C, lanes 1–6). It should be noted only one strong reactive protein band was observed reproducibly with the mouse p120-catenin 15D2 upon immunoprecipitation (Figure 3C). The faster migrating band that cross-reacted with FLαSH co-migrated with the protein band recognized by 15D2, and therefore these two antibodies were used interchangeably throughout the study. Taken together, these results show that Src-dependent phosphorylation is sufficient for WT-JMD degradation and Hakai-mediated ubiquitination, and that over-expression of Src and Hakai altered the equilibrium of WT-JMD stability by targeting more for degradation.

### Lysine Mutations Inhibit WT-JMD Degradation

As an independent test that WT-JMD instability was due to ubiquitin-dependent proteasomal degradation, we examined the JMD sequence for lysine residues that could be targets for ubiquitination. Two lysines are present within the JMD domain, and they were mutated to arginine (Figure 1A): lysine 5 (K5R-JMD) is located 17 amino acids upstream of the JMD octapeptide domain required for p120-catenin binding, and lysine 83 (K83R-JMD) is 52 amino acids downstream of the JMD octapeptide domain. Double mutations of both lysines (K5, 83R-JMD) were also examined. All constructs were transiently expressed in MDCK cells and analyzed using RFP fluorescence as a read-out of protein accumulation and stability.

**Figure 4:**
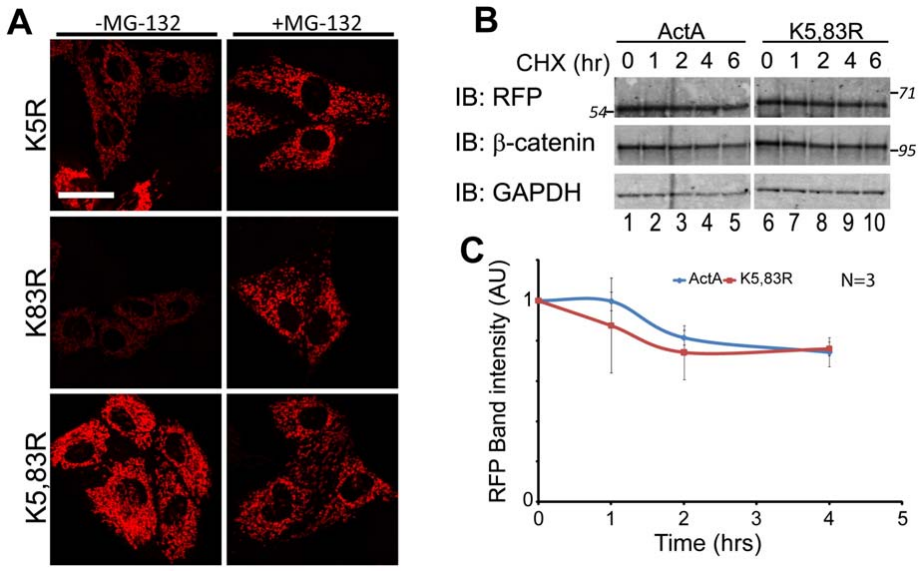
Lysine mutations inhibit WT-JMD degradation and differentially affect WT-JMD stability. (A) RFP immunofluorescence of MDCK cells transiently expressing K5R-JMD, K83R-JMD, or K5,83R-JMD under normal conditions (left column), or upon proteasome inhibition (+ MG-132). Images were processed under identical conditions and from the same experiment as images in Figure 1D. Scale bar is 25 µm. (B) MDCK cells were transiently transfected with ActA or K5,83R-JMD and incubated for 6 hours with cycloheximide, 40 hours post-transfection. Immunoblots (IB) were performed for RFP, β-catenin and GAPDH. Number(s) on the side of the gels are the apparent molecular weights of protein standards (× 10^∧^3). (C) Quantification of immunoblots from MDCK cells transiently expressing ActA or K5,83R-JMD and incubated for 6 hours with cycloheximide, 40 hours post-transfection. Band intensities from post nuclear supernatants were normalized to GAPDH to account for reduction in overall protein levels. Results are averaged from 3 independent experiments (+/− s.e.m.).

The level of K5R-JMD appeared greater than WT-JMD, but less than ActA (Figure 4A, compare with Figure 1D), while the level of K83R-JMD appeared slightly higher than WT-JMD (Figure 4A, compare with Figure 1D). The level of both K5R-JMD and K83R-JMD appeared to increase upon proteasome inhibition (Figure 4A). The level of the K5,83R-JMD double mutant was similar to ActA (Figure 4A, compare with Figure 1D), and did not appear to increase upon proteasome inhibition (Figure 4A). Comparison of RFP protein turnover in cells transiently expressing ActA or the lysine mutant K5,83R–JMD demonstrated that the level of the lysine mutants remaining (60%) as determined by RFP band intensities was similar to ActA (55%) (Figure 4B and C). These results demonstrate that K5 and K83 are required for ubiquitination and proteasomal degradation of JMD.

**Figure 5:**
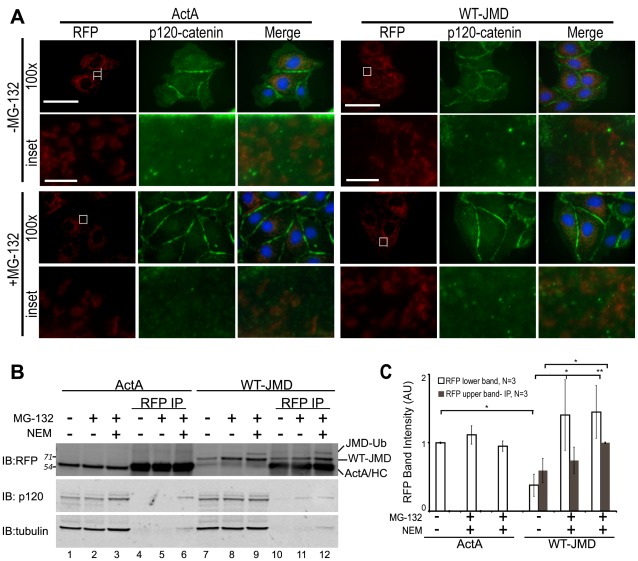
Localization and binding of WT-JMD and p120-catenin. (A) Immunofluorescence of MDCK cells transiently expressing ActA or WT-JMD. Images for RFP (red), p120-catenin (green) and merged are shown separately (100×). Boxed areas are shown as higher magnifications below (RFP-, p120- and merge-inset). All images were from the same experiment and processed identically between cell lines. Scale bar is 25 µm in 100× images, and 5 µm in insets. (B) Lysates and RFP immunoprecipitates (IP) of ActA and WT-JMD stable cell lines under normal conditions, upon proteasome inhibition, or NEM treatment (to inhibit de-ubiquitinating enzymes). Immunoblots (IB) for RFP show: a slower migrating band (upper band-JMD-Ub) that appears only in WT-JMD cells in the presence of MG-132 and NEM; the band identified as ActA/HC comprises a co-migrating ActA and the IgG heavy chain (HC). Number(s) on the side of the gels are the apparent molecular weights of protein standards (× 10^∧^3). (C) Quantification of RFP intensities normalized to tubulin in WT-JMD stables cell lines. Data averaged from 3 independent experiments (+/− s.e.m.), and 2 independently cloned stable cell lines; *p≤0.05, **p≤0.01.

### E-Cadherin Juxtamembrane Domain does not Recruit p120-Catenin to Mitochondria

p120-Catenin binds to the E-cadherin JMD [11], and is thought to be required for E-cadherin stability [12]. Since WT-JMD levels were low due to proteasomal degradation but could be stabilized by inhibition of the proteasome (Figure 2A), we analyzed whether p120-catenin localized with WT-JMD to mitochondria under either of these conditions. Surprisingly, p120-catenin did not co-localize with WT-JMD at mitochondria under either condition (Figure 5A). A very small amount of p120-catenin co-immunoprecipitated with WT-JMD when the proteasome was inhibited and WT-JMD accumulated (Figure 5B, lane 11). However, this amount was similar to background amounts of p120-catenin that co-immunoprecipitated with both ActA, which lacks a p120-catenin binding site, and WT-JMD upon inhibition of deubiquitinating enzymes (Figure 5B, lanes 6, 12). Note that the other catenins, β-catenin and α-catenin, also did not co-localize with WT-JMD as expected since only a truncated cytoplasmic domain of E-cadherin was expressed (Figure S3).

These results indicate that p120-catenin was not recruited to the E-cadherin JMD. Surprisingly, p120-catenin was also not recruited to the E-cadherin JMD when proteasomal degradation was inhibited. This result suggests that p120-catenin binding to WT-JMD is inhibited.

### JMD Lysine Mutations Differentially Affect p120-Catenin Localization

**Figure 6:**
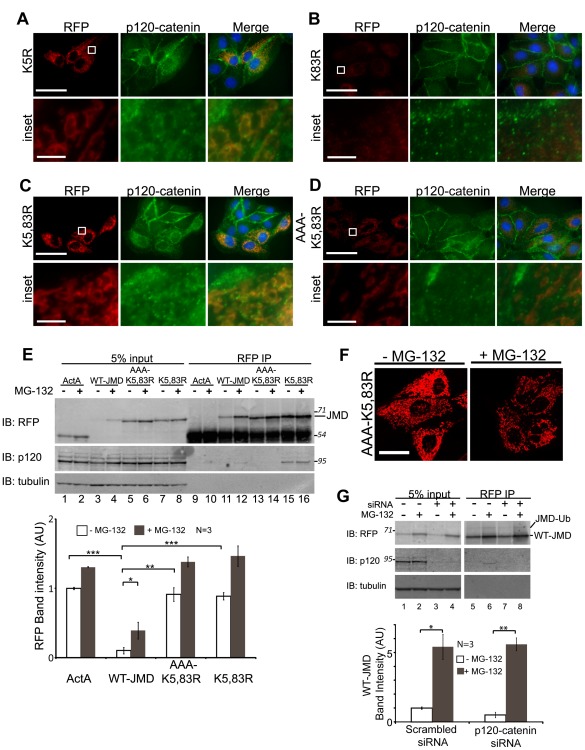
Lysine mutations differentially affect p120-catenin localization. (A-D) MDCK cells transiently expressing K5R-JMD. (A), K83R-JMD (B), K5,83R-JMD (C) or AAA-K5,83R-JMD (D). Cells were fixed and processed for immunofluorescence for RFP and p120-catenin. Boxed areas are shown at higher magnification below (inset). All images were from the same experiment and processed identically to those in Figure 5A. Scale bar is 25 µm in 100x images, and 5 µm in insets. (E) MDCK cells transiently expressing ActA, WT-JMD, K5,83R-JMD or AAA-K5,83R-JMD. Lysates and RFP immunoprecipitates (IP) were performed 40 hours post-transfection under normal conditions or upon proteasome inhibition. Immunoblots (IB) for RFP show similar levels of ActA, K5,83R-JMD and AAA-K5,83R-JMD levels. Immunoblots for p120-catenin show that p120-catenin is co-immunoprecipitated with K5,83R-JMD (lanes 15, 16) but not AAA-K5,83R-JMD (lanes 13, 14). Number(s) on the side are the apparent molecular weights of proteins standards (× 10^∧^3). Quantification of RFP immunoblots from cell lines transiently expressing ActA, WT-JMD, K5,83R-JMD or AAA-K5,83R-JMD. RFP levels for ActA, K5,83R-JMD and AAA-K5,83R-JMD were higher than WT-JMD. Data are averaged from 3 independent experiments (+/− s.e.m.); *p  = 0.031, **p<0.005, ***p≤0.001. (F) RFP immunofluorescence of MDCK cells transiently expressing AAA-K5,83R-JMD under normal conditions or upon proteasome inhibition (+MG-132). Images were taken and processed under identical conditions to each other and those in Figure 1D and Figure 4A. Scale bar is 25 µm. (G) MDCK cells stably expressing ActA or WT-JMD were transfected with either scrambled siRNAs (-) or p120-catenin specific siRNAs (+). Cells were extracted and RFP immunoprecipitations were preformed under normal conditions or upon proteasome inhibition (MG-132). Immunoblots (IB) were processed for RFP, p120-catenin and tubulin. Number(s) on the side of the gels are the apparent molecular weights of protein standards (× 10^∧^3). Quantification of WT-JMD levels upon knockdown of p120-catenin was normalized to tubulin. Results are averaged from 3 independent experiments (+/− s.e.m.); *p<0.05, **p<0.01.

In light of the fact that we could not find p120-catenin co-localized with, or bound to WT-JMD, we asked whether increased levels of the JMD lysine mutants were due to the lack of ubiquitination or p120-catenin binding. Significantly, p120-catenin clearly co-localized with K5R-JMD (Figure 6A) and K5,83R-JMD (Figure 6C), but not with K83R-JMD (Figure 6B). In addition, we noted a reduction in the localization of p120-catenin at cell-cell contacts in cells expressing K5,83R-JMD compared to non-transfected cells within the same image frame (Figure 6C).

RFP immunoprecipitation was used to quantify the levels of the JMD lysine mutants and examine p120-catenin binding. Transiently transfected MDCK cells were extracted in the presence or absence of proteasome inhibition. The level of WT-JMD increased 4-fold upon inhibition of the proteasome, as expected (Figure 6E, lanes 3, 4; Figure 6E-graph; *p = 0.001). The levels of ActA and the K5,83R-JMD mutant were similar and only increased 0.3-fold (p = 0.0008) and 0.6-fold (p = 0.026), respectively, upon proteasome inhibition (Figure 6E, lanes 1, 2 and 7, 8; Figure 6E-graph). The difference between the levels of ActA, K5,83R-JMD and WT-JMD was statistically significant in all cases (***p≤0.001). As expected, we detected background levels of p120-catenin co-immunoprecipitated with WT-JMD in the presence of proteasome inhibition compared to ActA (Figure 6E, lane 12). However, we detected a 10-fold higher amount of p120-catenin bound to K5,83R-JMD compared to WT-JMD, and this amount increased only 0.4 fold upon proteasome inhibition (Figure 6E, lanes 11, 12 vs. 15, 16). Furthermore, we observed p120-catenin/RFP co-localization in MDCK cells transiently expressing either K5R-JMD (Figure S4A) or K5,83R-JMD (Figure S4C), but not K8R-JMD (Figure S4B).

### p120-Catenin Binding is not Responsible for Stability of the JMD Lysine Mutants

Although the data thus far indicate a correlation between increased stability of JMD and increased p120-catenin binding to the JMD, it is also possible that increased JMD stability is due to the lack of ubiquitination. To test whether the increased level of K5,83R-JMD was due to loss of ubiquitination or increased binding of p120-catenin, we mutated the binding site for p120-catenin [12] to AAA in the K5,83R-JMD mutant (AAA-K5,83R-JMD; Figure 1A). Western blots of whole cell lysates revealed that the level of AAA-K5,83R-JMD increased slightly upon proteasome inhibition (0.5-fold; p  = 0.021), similar to that of ActA and K5,83R-JMD under the same conditions (Figure 6E, lanes 5, 6; 6E-graph), and was greater than WT-JMD (p  = 0.004). While p120-catenin colocalized with K5,83R-JMD, it did not co-localize with AAA-K5,83R-JMD (Figure 6D). Furthermore, western blots of RFP immunoprecipitates revealed that p120-catenin was not bound to AAA-K5,83R-JMD (Figure 6E, lanes 13, 14). It should also be noted p120-catenin did not colocalize with RFP in MDCK cells transiently expressing AAA-K5,83R-JMD (Figure S4D).

These results indicate that K5R-JMD contains a mutation in the primary ubiquitination site, and that mutation of K5 within WT-JMD allows the recruitment of p120-catenin to the JMD.

### p120-Catenin Knockdown has a Limited Affect on JMD Degradation

To test directly if p120-catenin affects WT-JMD levels, we depleted p120-catenin by ∼95% using specific siRNAs compared to p120-catenin levels in WT-JMD and ActA stable cells treated with scrambled siRNAs (Figure 6G; A. Hartsock, unpublished data). The level of WT-JMD in p120-catenin knockdown cells was 65% less than that in controls (p  = 0.065). In the presence of proteasome inhibition, there was a significant increase in WT-JMD levels in both p120-catenin knockdown (**p  = 0.008) and control cells (*p  = 0.020) (Figure 6G and graph). There was no affect on ActA levels by p120-catenin knockdown (A. Hartsock, unpublished data). These results indicate that accumulation of WT-JMD upon proteasome inhibition was independent of p120-catenin expression.

### Lysine Mutations in Full Length E-cadherin and Mitochondrial Targeted E-cadherin JMD have Similar Effects on Protein Accumulation

**Figure 7:**
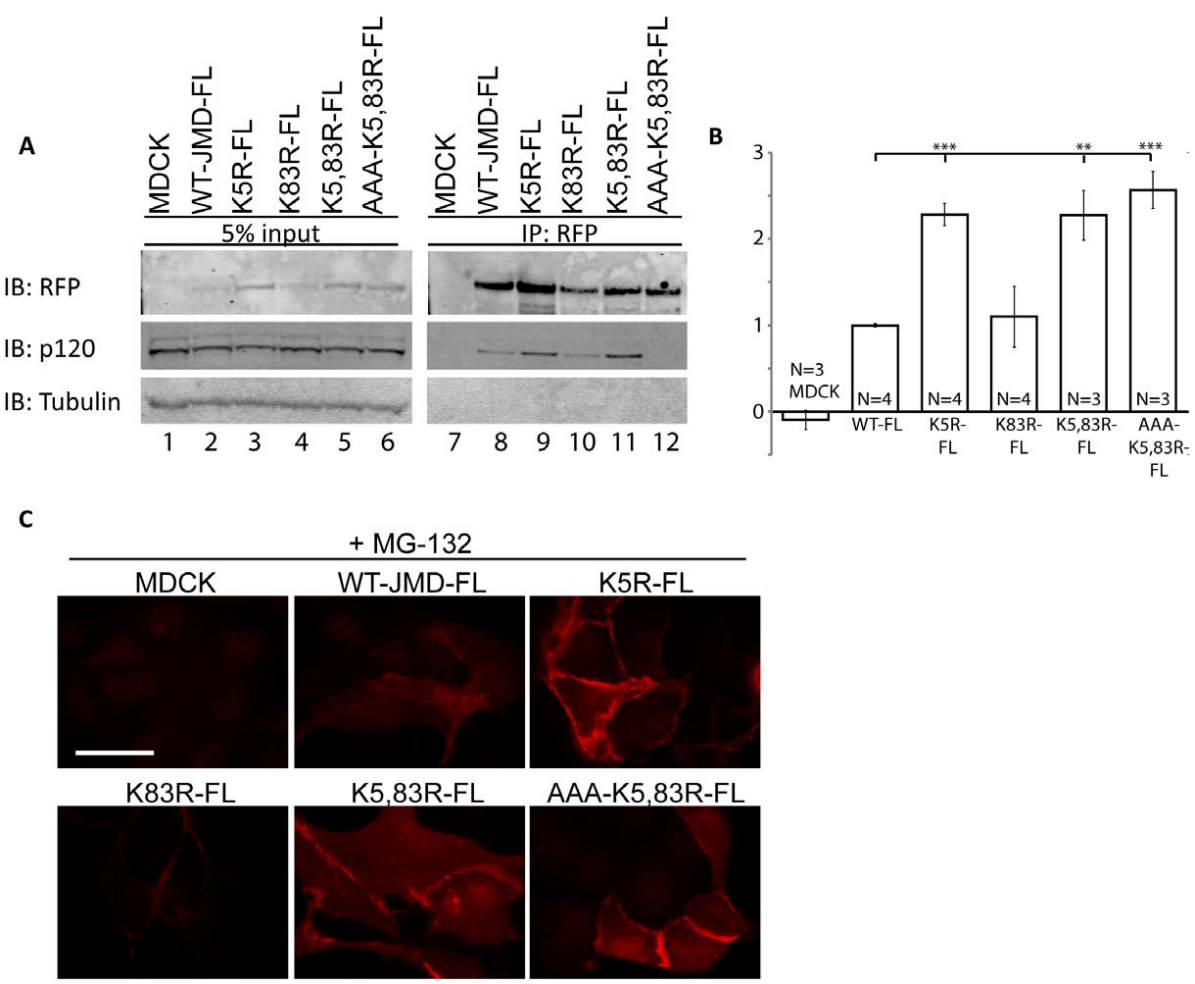
Analysis of full length E-cadherin lysine mutants verifies results of RFP-JMD mitochondrial degradation assay. (A) Parental MDCK cells and MDCK cells transiently expressing full length E-cadherin RFP fusion proteins (WT-FL), or full length E-cadherin RFP fusion proteins containing lysine mutant sequences (K5R-FL; K83R-FL; K5,83R-FL; AAA-K5,83R-FL). RFP immunoprecipitates were performed 40 hours post-transfection under normal conditions. Immunoblots (IB) for RFP show higher levels of K5R-FL, K5,83R-FL and AAA-K5,83R-FL compared to WT-FL. Immunoblots for p120-catenin show that p120-catenin is co-immunoprecipitated with all E-cadherin mutants, except AAA-K5,83R-FL. (B) Quantification of levels of transiently expressed E-cadherin RFP fusion proteins in MDCK cells. RFP band intensities were normalized to tubulin. Results are averaged from 3–4 different experiments as indicated (+/− s.e.m.); ^∧^ p<0.03, *p<0.01, **p<0.001, ***p<0.0001. (C) RFP immunofluorescence of MDCK cells transiently mock transfected, or expressing WT-JMD-FL, K5R-WT-FL, K83R-FL, K5,83R-FL or AAA-K5,83R-FL. All images were taken in cells in which the proteasome was inhibited (+MG-132) to ensure visualization of RFP in WT-JMD-FL and K83R-FL transiently expressing cell lines. Images were taken and processed under identical conditions to each other. Scale bar is 25 µm.

To rule out the possibility that the effects of ubiquitination and p120-catenin binding on JMD levels were due to mitochondrial targeting, we transiently expressed full length E-cadherin tagged with RFP in which the JMD was either wild-type sequence (WT-FL) or contained lysine mutations (K5R-FL; K83R-FL; K5,83R-FL; AAA-K5,83R-FL); the RFP fusion proteins were isolated by RFP immunoprecipitation from whole cell lystates (Figure 7A). The levels of K5R-FL, K5,83R-FL and AAA-K5,83R-FL were twice the levels of WT-FL and K83R-FL. In addition, the level of K5R-FL was similar to K5,83R-FL and AAA-K5,83R-FL (Figure 7A, lanes 3, 5 and 6; Figure 7B), while K83R-FL levels were similar to WT-FL (Figure 7A, lanes 2, 4; Figure 7B). p120-Catenin did not co-immunoprecipitate with AAA-K5,83R-FL similar to mitochondrial targeted AAA-K5,83R-JMD (Figure 7A, lane 12). Furthermore, we observed RFP immunofluorescence in MDCK cells either mock transfected or transiently expressing full length E-cadherin tagged with RFP in which the JMD was either wild-type (WT-FL) or contained lysine mutations (K5R-FL; K83R-FL; K5,83R-FL; AAA-K5,83R-FL) (Figure 7C). To detect transiently expressed WT-JMD-FL and K83R-FL the proteasome was inhibited. Note these constructs localized at cell cell contacts (Figure 7C). Together these data verify our results and conclusions on the role of ubiquitination and p120-catenin binding in the degradation of mitochondrial targeted E-cadherin JMD.

## Discussion

Plasma membrane protein levels are regulated by post-translational modifications such as ubiquitination, and phosphorylation [2], [3], [4], [5]. Here, we investigated the roles of ubiquitination, phosphorylation and p120-catenin binding on targeting E-cadherin for degradation. The results provide new insight into the regulation of E-cadherin levels at the plasma membrane.

p120-Catenin binds the JMD in the cytoplasmic domain of E-cadherin, and is required for E-cadherin stability at the plasma membrane [12], although the precise role of p120-catenin in E-cadherin stabilization is unclear. The JMD domain also binds to, and is ubiquitinated by the E3 ligase Hakai in a Src-dependent manner, which results in internalization of E-cadherin from the plasma membrane [17]. It is unclear whether or not JMD binding to p120-catenin and JMD ubiquitination are mechanistically linked, and whether pathways controlling internalization and degradation are similar or different. Here, we targeted E-cadherin JMD to mitochondria to isolate JMD protein-protein interaction and protein modifications *in vivo* that were dependent upon JMD recruitment of p120-catenin, Hakai and Src to the mitochondria. Importantly, we found little or no affect of expression of mitochondrial targeted WT-JMD on endogenous E-cadherin, β-catenin and α-catenin levels, nor the organization or migration of an epithelial sheet. Therefore, this approach did not appear to affect the cell generally, and thus could be used as an *in vivo* assay for JMD binding to p120-catenin compared to JMD ubiquitination, and the consequences on JMD degradation.

Although p120-catenin binds to a minimal octapeptide sequence within E-cadherin JMD [11], we found that WT-JMD did not bind p120-catenin, despite a large cytoplasmic pool of p120-catenin, and was degraded rapidly. Proteasome inhibition resulted in WT-JMD accumulation, but p120-catenin was neither recruited to mitochondria, nor co-immunoprecipitated with WT-JMD. While it is possible that the RFP tag on the JMD could inhibit p120-catenin binding, we found p120-catenin was recruited and co-immunoprecipitated with mitochondrial targeted K5R-JMD and K5,83R-JMD mutants and all full length E-cadherin RFP fusion proteins. Thus, p120-catenin recruitment and binding can occur in the presence of the RFP tag. It is more likely that p120-catenin binding to WT-JMD was inhibited by a modification(s) that targeted WT-JMD for degradation by the proteasome.

When we examined WT-JMD for ubiquitination in the presence of NEM or Ub-HA, we detected a small amount of ubiquitinated protein (JMD-Ub), which was sensitive to the deubiquitination enzyme Usp2. The bulk of WT-JMD under any of these conditions did not appear to be ubiquitinated but still did not bind p120-catenin. We suggest that WT-JMD was ubiquitinated in cells, but deubiquitinating enzymes were released upon cell lysis and deubiquitinated most of the WT-JMD, even in the presence of NEM. We cannot rule out other modifications of JMD such as tyrosine phosphorylation that might have additional effects on sterically inhibiting JMD/p120-catenin binding.

Significantly, p120-catenin was recruited to the JMD that had mutations of specific lysine residues (K5R-JMD and K5,83R-JMD). These mutations blocked JMD degradation and resulted in JMD accumulation. We showed that K5R-JMD was sufficient to inhibit proteasomal degradation of JMD, and result in p120-catenin binding to either mitochondrial targeted JMD or full length E-cadherin K5R mutants. An additional mutant AAA-K5,83R deleted the p120-catenin binding site in combination with both lysine mutations and also resulted in accumulation of JMD, but, as expected, did not recruit p120-catenin. Therefore, stabilization and accumulation of JMD was conferred by the lysine mutations that resulted in inhibition of JMD degradation, and not binding of p120-catenin.

The K5R mutation is 17 amino acids upstream of the p120-catenin binding site and increased the stability of the JMD to a greater extent than K83R, which is closer to the β-catenin binding domain of the E-cadherin cytoplasmic tail. We suggest that p120-catenin binding to JMD might, therefore, depend on the state of K5 ubiquitination; the lack of ubiquitination in K5R mutants allows p120-catenin binding, whereas the addition of an ubiquitin to wild-type K5 might sterically inhibit p120-catenin binding. Further *in vitro* studies of JMD and p120-catenin binding will be needed to fully test this possibility.

Previous studies expressed a truncated E-cadherin containing the extracellular domain and only the first 15 amino acids of the JMD, which included K5. This mutant E-cadherin did not accumulate within the plasma membrane, but instead colocalized with endocytic markers [23]. However, this mutant E-cadherin did not contain the octapeptide required for E-cadherin/p120-catenin binding [11], or the two tyrosine residues required to stabilize E-cadherin/Hakai binding [17]. Hence it is not possible to draw conclusions about the role of p120-catein or Hakai binding on the distribution of that mutant E-cadherin.

It has been proposed that the E3 ligase Hakai ubiquitinates E-cadherin in a Src-dependent manner and p120-catenin is not bound during this process [17]. However, it was not shown directly whether Hakai or Src activity and subsequent JMD ubiquitination affected JMD/p120-catenin binding. While we cannot rule out that Src over-expression directly affected the state of p120-catenin phosphorylation and hence increased the degradation of WT-JMD, we demonstrated that increased expression of Src-GFP or Hakai-FLAG alone increased WT-JMD degradation. Furthermore, the results of our E-cadherin JMD degradation assay showed that over-expression of either Src or Hakai resulted in increased ubiquitination and degradation of WT-JMD.

These data, combined with p120-catenin binding to mutant K5R-JMD, further indicate that once ubiquitination of K5 has occurred p120-catenin cannot bind to E-cadherin and thereby confer stability to E-cadherin. On the other hand, binding of p120-catenin to the JMD might mask the primary ubiquitination site in E-cadherin (K5) thereby preventing ubiquitination, internalization and degradation. Since Hakai binds E-cadherin JMD [17], our data support the hypothesis that Hakai may be in competition with p120-catenin for binding the JMD. Further *in vitro* binding studies will be needed to test this hypothesis directly.

With the close proximity of p120-catenin and ubiquitination sites in the JMD it is difficult to determine exactly how p120-catenin confers stability on E-cadherin. The E-cadherin-JMD/p120-catenin binding interface includes a static region of protein-protein interaction that contains the highly conserved octapeptide sequence, and a more dynamic region upstream containing K5 [24]. It was proposed the N-terminal of p120-catetin interacts weakly with this dynamic region of E-cadherin JMD thereby competing binding with clathrin-mediated endocytic machinery. Furthermore, it was suggested this interaction might be reduced by phosphorylation of p120-catenin, which might further expose conserved tyrosine residues in the E-cadherin JMD to Src kinase [24]. In our studies, however, we showed that mutations of both K5 and K83 inhibited E-cadherin degradation even in the absence of p120-catenin binding (AAA-K5,83R). In addition, we showed that WT-JMD accumulated upon proteasome inhibition in cells in which p120-catenin levels were reduced ∼95%. Combined, our results indicate p120-catenin binding is not involved in WT-JMD ubiquitin-dependent degradation. Furthermore, our analysis of the K5 and K83 mutations in full length E-cadherin indicate this mechanism is involved in maintaining the equilibrium of E-cadherin localization and stability at the plasma membrane. Our results indicate that E-cadherin can be mono- and poly-ubiquitinated, and it is possible that both combination of ubiquitination are responsible for targeting E-cadherin for proteasomal degradation.

Our results support the hypothesis that competitive binding between p120-catenin and modifications of E-cadherin by Hakai (ubiquitination) or Src (tyrosine phosphorylation) at the JMD regulate E-cadherin degradation. While it is possible that p120-catenin binding to the JMD may inhibit Src or Hakai binding and hence ubiquitination and degradation, our results show that ubiquitination on at least K5 inhibits p120-catenin binding, and results in proteasomal degradation of E-cadherin. In a broader context, ubiquitination of E-cadherin JMD may block or even displace p120-catenin, resulting in E-cadherin internalization from the plasma membrane and rapid proteasomal degradation.

## Materials and Methods

### cDNA Constructs

ActA-RFP [18] was used as a control. WT-JMD was created by sub-cloning mouse E-cadherin JMD from the cytoplasmic domain of E-cadherin [25] into β-catenin-ActA [18] using EcoRI at the 3′ end and BamHI at the 5′ end (FP:GGGAATTCCGCCACCA-TGCGGTACCTCAGAAC; RP:CCGGATCCCCTCCTCCTGCCGTGGGGTCG). Mutations were made using Stratagene QuickChange® site directed mutagenesis kit on the base construct WT-JMD. All reverse primers were the exact reverse compliment of the forward primers. K5R: AGAA-CGGTGGTCAGAGAGCCCCTGCTGCCACCAGA, K83R: GATGAAAACCTGAGG-GCAGCC-GACAGCGACCCC. Double mutations were made in tandem. AAA-K5,83R was made using K5,83R as a base construct (AAA FP: GGAGGTGGAGCAGCAGCC-CAGGACTTTGATTTGAGC). All mutations were verified by sequencing. Ub-HA was a gift from Dr. Z. Chen (University of Texas Southwestern Medical Center, Dallas, TX). Src-GFP was a gift from Dr. Michael Way (Cancer Research, London, UK). Hakai-FLAG was a gift from Dr. Y. Fujita (University College London, UK).

### Stable Cell Lines

Cell lines were maintained in Dulbecco’s modified Eagle’s medium (DMEM; Life Technologies) supplemented with 10% FBS (Cell Generations). MDCK cells [20] were transfected with ActA or WT-JMD plasmid using LipofectAMINE 2000 (Invitrogen) according to the manufacturer’s instructions as described previously [18]. Cells were sorted for RFP expression using Fluorescence Activated Cell Sorting (FACS) to increase the number of positive RFP cells, and then selected with 0.3 mg/ml of G418 for 10–14 days. Cells positive for RFP were plated at single cell density, and single cell clones were isolated using cloning rings, expanded and replicates were frozen and stored in liquid nitrogen. The use of DNA and generation of cell lines is approved by Stanford APLAC (Administrative Panel on Laboratory Animal Care).

### Transient Transfections

MDCK cells were transfected with plasmids (ActA, WT-JMD, K5R-JMD, K83R-JMD, K5,83R-JMD, and AAA-K5,83R-JMD) using LipofectAMINE 2000 (Invitrogen) according to manufacturer’s instructions. Cells were plated for 40 hours prior to experiments. All transiently transfected cell lines were transfected on the same day and time for direct comparison between all cell lines. Ub-HA, Hakai-FLAG and Src-GFP were transfected into WT-JMD cell line. Mock transfections were performed with transfection reagents only.

### siRNA

MDCK cells were incubated with siRNAs using LipofectAMINE 2000 (Invitrogen). Scrambled siRNA was siGENOME non-targeting siRNA #3 (Dharmacon siRNA Technologies; catalog # D-001210-03), p120-Catenin siRNA utilized the sequence CAGUCAAGAAAGUG-GUGAAUU (Dharmacon siRNA Technologies). 100 ng of siRNA was transfected with 10 ul of lipofectAMINE in a final volume of 200 ul in reduced serum media. Medium was replaced 24 hours post-transfection with DMEM, 10% FBS without antibiotics.

### Immunofluorescence Microscopy

Stable cell lines were plated on collagen I-coated coverslips and grown for 40 hours. Transiently transfected cells were plated on collagen I-coated coverslips 6 hours post-transfection and grown for 40 hours. Coverslips were rinsed with PBS-ME and fixed in 3% paraformaldehyde in PBS-ME for 13 minutes at room temperature (RT). After washing, cells were incubated in 0.5% saponin in PBS-ME for 15 minutes, followed by blocking buffer (1% goat serum, 0.1% saponin in PBS-ME) for 1 hr at RT. Cells were incubated with primary antibodies in blocking buffer overnight at 4°C, washed in PBS-ME, incubated with the secondary antibody for 30 min at RT, and washed in PBS-ME. The following antibodies were used: rabbit RFP (Rockland; catalog # 600-401-379; 1∶500); p120-catenin (15D2; gift from Dr. Al Reynolds, Vanderbilt University; 1∶750); mouse E-cadherin antibody raised to extracellular domain (mRR1; cells were gift from Dr. Barry Gumbiner, University of Virginia; 1∶100); mouse ZO-1 (Zymed; catalog # 33–9100; 1∶500); mouse vinculin (Sigma; catalog # V4505; 1∶100); mouse tubulin (Sigma; catalog # T6199; 1∶1000); mouse β-catenin (Transducin; catalog # C19220; 1∶500); mouse α-catenin (Alexis Corp; catalog # 804-101-C100; 1∶100). Secondary antibodies used were FITC-conjugated goat anti-mouse antibody (Jackson ImmunoResearch; catalog # 111-295-144; 1∶100) and Rhodamine-conjugated goat anti-rabbit antibody (Jackson ImmunoResearch; catalog # 115-095-146; 1∶100). Cells were mounted in Vectashield containing DAPI. Images of antibody-stained cells were taken using a Zeiss Axiovert 200 microscope and Axiovision software (Carl Zeiss Micro-Imaging, Inc.). All images within an experiment were taken using the same exposure on the same day. Images were adjusted identically within the same experiment using Adobe Photoshop to optimize image quality.

### Cell Extraction/Immunoprecipitations

All extractions were performed at 4°C. Cells were washed twice in TBS. Cells were extracted with 0.1% Triton X-100 in CSK buffer (50 mM NaCl, 300 mM sucrose, 10 mM Pipes, pH 6.8, 3 mM MgCl_2_) with 0.1 ug/ml Pefabloc. RFP containing protein chimeras were immunoprecipitated using a rabbit polyclonal RFP antibody at 1∶100 dilution (Clonetech; catalog # 632397). E-cadherin immunoprecipitations used E2 antibody raised against the cytoplasmic domain of E-cadherin [26]. Cells were incubated with 10 µM MG-132 for 4 hours prior to extraction. 100 µM cycloheximide (CHX) was added to cell culture media for up to 6 hours prior to extraction. NEM was resuspended in ethanol and used at 10 mM final concentration in extraction buffer; control extractions were performed using an equivalent volume of ethanol. For Usp2 treatments of immunoprecipitates, antibody-protein complexes isolated on Protein A beads in CSK buffer were incubated with Usp2 (5 µg- gift from Dr. Ron Kopito, Stanford University) at 37°C for 30 minutes. 1 mM DTT was added to beads after the final wash in CSK buffer prior to incubation of Usp2 to neutralize residual NEM activity. Proteins were processed for SDS-PAGE, separated in BioRAD 3-8% Tris Acetate gradient gels, and transferred overnight to PVDF membranes.

### Western Blot Analysis

PVDF membranes were incubated in blocking buffer (5% milk, 2% BSA, 1% goat serum in TBS) for 1 hr at RT. Blots were incubated with antibodies overnight at 4°C. The following antibodies were used: rabbit RFP (Rockland; catalog # 600-401-379; 1∶500), p120-catenin (15D2; gift from Dr. Al Reynolds, Vanderbilt University; 1∶500), mouse tubulin (Sigma; catalog # T6199; 1∶2000), mouse β-catenin (Transducin; catalog # C19220; 1∶500), mouse HA.11 (Covance; catalog # MMS-101P-500), mouse GFP (Clonetech; catalog # 623375; 1∶100). Secondary antibodies used were goat anti mouse 800CW (1∶15,000, LI-COR Biosciences) and goat anti-rabbit Alexa Fluor (1∶15,000, Invitrogen). Membranes were scanned using an Odyssey imager and software (LI-COR Biosciences) and bands were quantified using Image J. Resulting images of entire blots were adjusted using Adobe Photoshop and cropped only after all adjustments were performed. In all cases an n of 1 is equivalent to analysis from 1 gel and subsequent blot.

### Wound Healing Assay

Cells were trypsinized and resuspended in low calcium media (10% FBS, 5 uM Ca ^2+^ in DMEM) and plated to confluency on collagen I-coated 35 mm glass bottom dishes (MatTek) for 75 minutes. Medium was replaced with DMEM containing 1.8 mM Ca^2+^ and incubated for 3hours. A single scratch was made along the length of the dish using a 1 ml pipette tip and the media was replaced with imaging media (10% FBS, 25 mM Hepes, in DMEM without phenol red). Cells were imaged at 10X magnification every 15 min for 15 hours at 37°C on an inverted microscope (Axiovert 200 M) controlled with Slidebook software (Intelligent Imaging Innovations); 5 sites were imaged along the wound edge. The total wound area covered was quantified using Image J software. Time zero was 15 min post-scratch.

## Supporting Information

Figure S1 **Expression of WT-JMD does not affect protein localization or cell organization. (A)** Immunofluorescence images of MDCK stably expressing ActA or WT-JMD. Cells were fixed and labeled for E-cadherin, ZO-1, vinculin, tubulin, phalloidin (green) and RFP (red). All images were from the same experiment and processed identically between cell lines. Scale bar is 25 µm in 100X images. **(B)** Still images of MDCK cells and MDCK cells stably expressing either ActA or WT-JMD at 0 and 15 hours of wound healing assays. Scale bar is 100 µm in 10X images **(C)** Quantification of total area covered during 15 hours of a wound healing assay of MDCK cells, and MDCK cells stably expressing either ActA or WT-JMD.
(TIF)

Figure S2 **ActA and WT-JMD localize at mitochondria. (A)** Endogenous RFP fluorescence of MDCK cells stably expressing ActA or WT-JMD under normal conditions and upon proteasome inhibition. Mitotracker was used to identify mitochondria. Images were taken on the same day at the same exposure, and brightness and contrast were adjusted identically between cell lines to demonstrate locazlization of RFP with mitochondria. Scale bar is 25 µm in 100X images.
(TIF)

Figure S3 β**- and** α**-Catenin do not localize at mitochondria. (A-B)** β-catenin (A-green) or α-catenin (B-green) and RFP (red) immunofluorescence images of MDCK cells stably expressing ActA or WT-JMD under normal conditions and upon proteasome inhibition. Images were taken and processed under identical conditions. Scale bar is 25 µm in 100X images.
(TIF)

Figure S4 **Lysine Mutations Differentially Affect p120-catenin localization. (A-D)** MDCK cells were transiently transfected with either K5R-JMD (A), K83R-JMD (B), K5,83R-JMD (C) or AAA-K5,83R-JMD (D). Cells were fixed and processed for immunofluorescence for RFP and p120-catenin. Insets are cropped area denoted in 100X images (A, C). All images were from the same experiment and processed identically. Scale bar is 25 µm in 100X images and 5 µm in insets.
(TIF)
