# Supplementary figures and images for: Competitive Regulation of E-Cadherin JuxtaMembrane Domain Degradation by p120-Catenin Binding and Hakai-Mediated Ubiquitination
Source: PLoS One. 2012 May 31;7(5):e37476. doi: 10.1371/journal.pone.0037476 (PMC3365061; doi:10.1371/journal.pone.0037476)

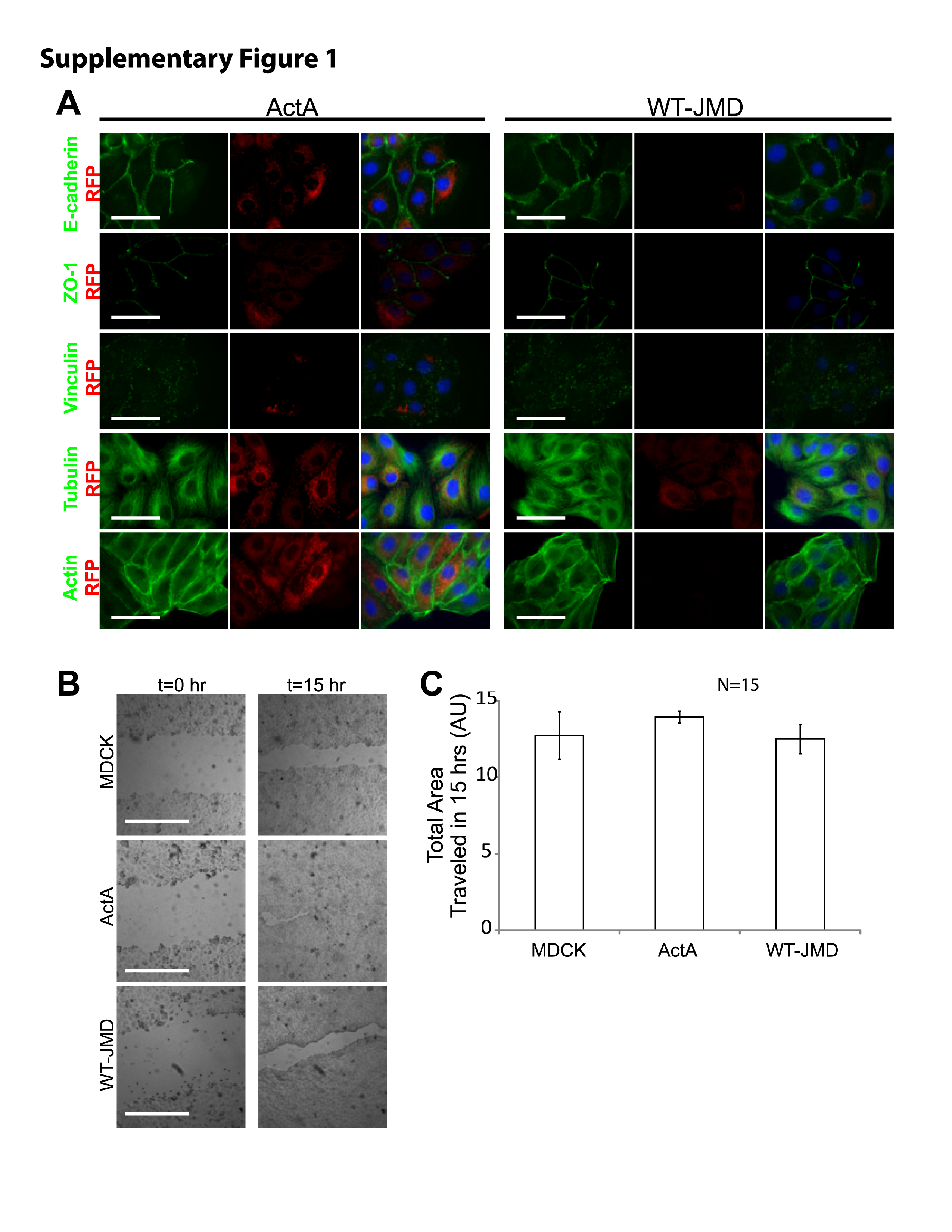

Supplement: Figure S1 — Expression of WT-JMD does not affect protein localization or cell organization. (A) Immunofluorescence images of MDCK stably expressing ActA or WT-JMD. Cells were fixed and labeled for E-cadherin, ZO-1, vinculin, tubulin, phalloidin (green) and RFP (red). All images were from the same experiment and processed identically between cell lines. Scale bar is 25 µm in 100X images. (B) Still images of MDCK cells and MDCK cells stably expressing either ActA or WT-JMD at 0 and 15 hours of wound healing assays. Scale bar is 100 µm in 10X images (C) Quantification of total area covered during 15 hours of a wound healing assay of MDCK cells, and MDCK cells stably expressing either ActA or WT-JMD. (TIF) [file pone.0037476.s001.tif]

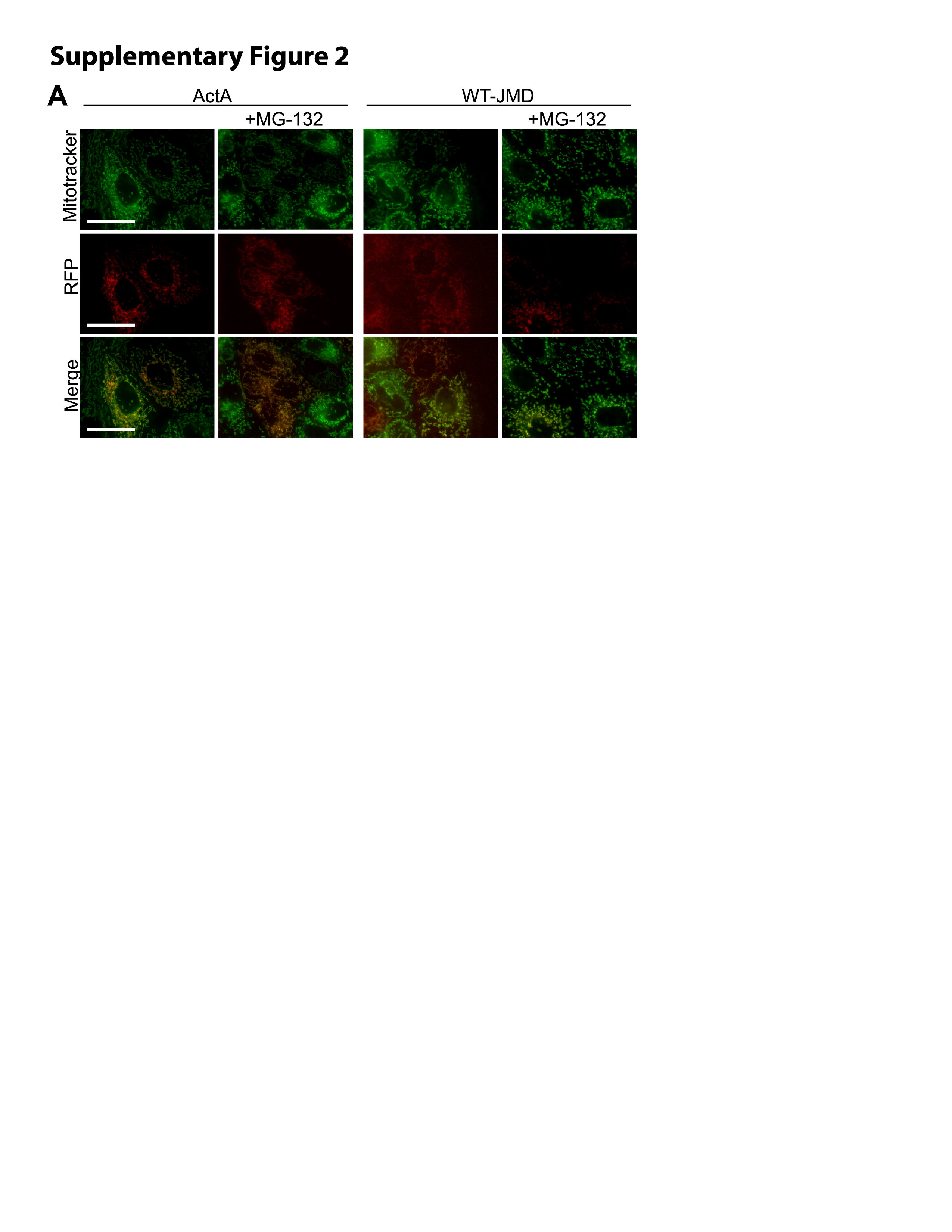

Supplement: Figure S2 — ActA and WT-JMD localize at mitochondria. (A) Endogenous RFP fluorescence of MDCK cells stably expressing ActA or WT-JMD under normal conditions and upon proteasome inhibition. Mitotracker was used to identify mitochondria. Images were taken on the same day at the same exposure, and brightness and contrast were adjusted identically between cell lines to demonstrate locazlization of RFP with mitochondria. Scale bar is 25 µm in 100X images. (TIF) [file pone.0037476.s002.tif]

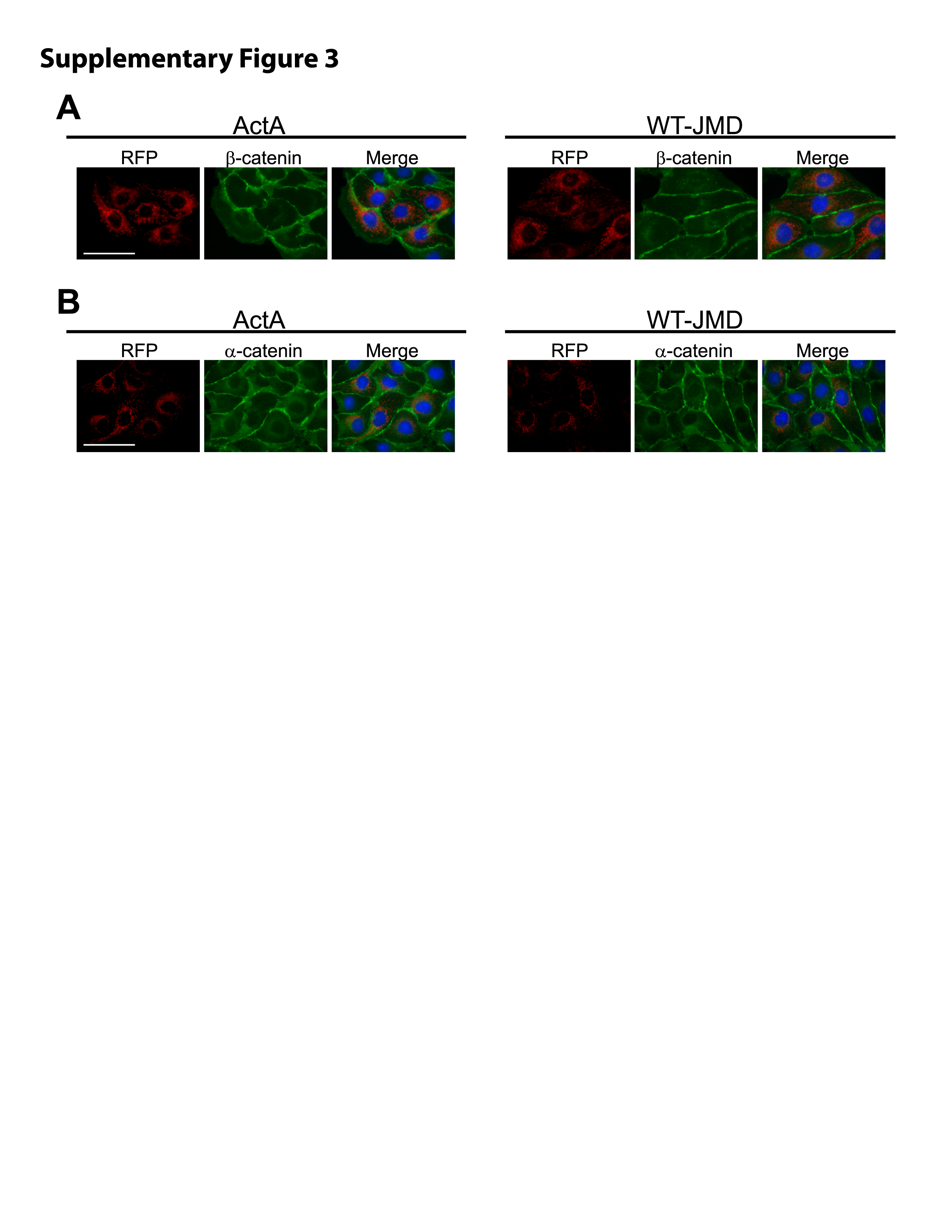

Supplement: Figure S3 — β- and α-Catenin do not localize at mitochondria. (A-B) β-catenin (A-green) or α-catenin (B-green) and RFP (red) immunofluorescence images of MDCK cells stably expressing ActA or WT-JMD under normal conditions and upon proteasome inhibition. Images were taken and processed under identical conditions. Scale bar is 25 µm in 100X images. (TIF) [file pone.0037476.s003.tif]

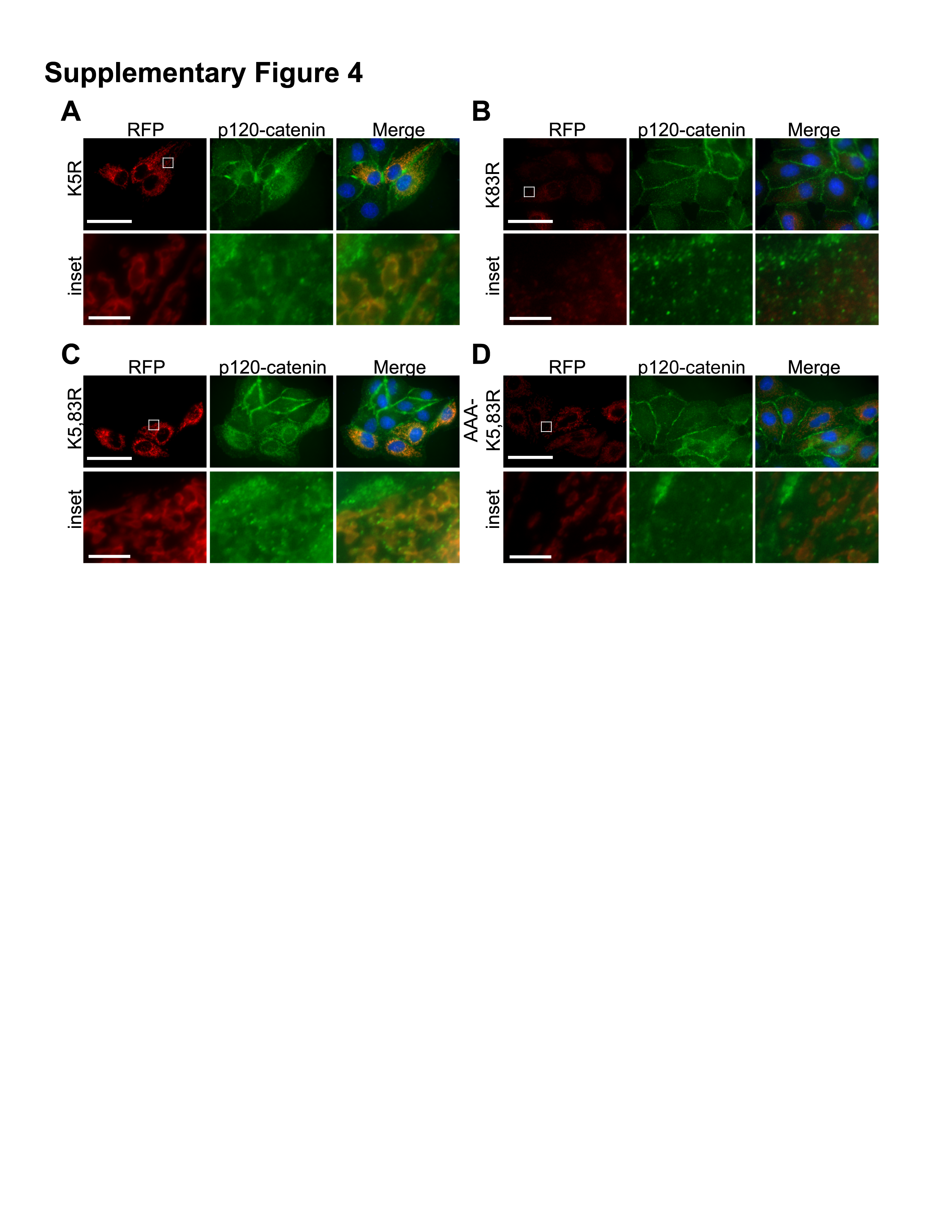

Supplement: Figure S4 — Lysine Mutations Differentially Affect p120-catenin localization. (A-D) MDCK cells were transiently transfected with either K5R-JMD (A), K83R-JMD (B), K5,83R-JMD (C) or AAA-K5,83R-JMD (D). Cells were fixed and processed for immunofluorescence for RFP and p120-catenin. Insets are cropped area denoted in 100X images (A, C). All images were from the same experiment and processed identically. Scale bar is 25 µm in 100X images and 5 µm in insets. (TIF) [file pone.0037476.s004.tif]
